# Combination of uniform design with artificial neural network coupling genetic algorithm: an effective way to obtain high yield of biomass and algicidal compound of a novel HABs control actinomycete

**DOI:** 10.1186/1475-2859-13-75

**Published:** 2014-05-24

**Authors:** Guanjing Cai, Wei Zheng, Xujun Yang, Bangzhou Zhang, Tianling Zheng

**Affiliations:** 1 State Key Laboratory of Marine Environmental Science and Key Laboratory of MOE for Coast and Wetland Ecosystems, School of Life Sciences, Xiamen University, No. 422, Siming Nan Road, Xiamen 361005, China; 2 ShenZhen Research Institute of Xiamen University, ShenZhen 518057, China

**Keywords:** Novel algicidal actinomycete, Uniform design, Artificial neural network coupling genetic algorithm, High yield of biomass and algicidal compound, HABs control

## Abstract

Controlling harmful algae blooms (HABs) using microbial algicides is cheap, efficient and environmental-friendly. However, obtaining high yield of algicidal microbes to meet the need of field test is still a big challenge since qualitative and quantitative analysis of algicidal compounds is difficult. In this study, we developed a protocol to increase the yield of both biomass and algicidal compound present in a novel algicidal actinomycete *Streptomyces alboflavus* RPS, which kills *Phaeocystis globosa*. To overcome the problem in algicidal compound quantification, we chose algicidal ratio as the index and used artificial neural network to fit the data, which was appropriate for this nonlinear situation. In this protocol, we firstly determined five main influencing factors through single factor experiments and generated the multifactorial experimental groups with a U_15_(15^5^) uniform-design-table. Then, we used the traditional quadratic polynomial stepwise regression model and an accurate, fully optimized BP-neural network to simulate the fermentation. Optimized with genetic algorithm and verified using experiments, we successfully increased the algicidal ratio of the fermentation broth by 16.90% and the dry mycelial weight by 69.27%. These results suggested that this newly developed approach is a viable and easy way to optimize the fermentation conditions for algicidal microorganisms.

## Background

With the increasing influence of human activity, harmful algal blooms, also sometimes known as red tides, have happened more frequently and severely [1-3]. The tremendous accumulation of algal cells destroys the natural harmony of the ocean environment by discoloring the water, disrupting food-web dynamics, depleting oxygen and even poisoning the other creatures [4,5]. Many approaches have been tried [6-8], and the limitation of physical and chemical methods [9] has made biological control the research hotspot. The bacteria-algae interaction plays an important role in both enhancing and decreasing algal blooms *in situ*[10] and, with the discovery of numerous bacterial strains exhibiting strong and specific algicidal activity, provides a potential cheap, efficient and environmentally-friendly way to terminate the blooms or even prevent their occurrences [11-15]. Although the discovery of algicidal bacterium could be traced to 1925 [16], there are still few reports about microbial control of red tides in field tests [17]. An inevitable problem concerns how to bring the algicidal microbes into the application stage with the help of mature fermentation technologies.

Most algicidal microbes affect the growth of algae through the secreted metabolites. These algicidal metabolites might be proteins, peptides, amino acids, bio-surfactants, and antibiotics [18]. Better understandings of the algicidal microbes require systematic studies about the chemical nature of these compounds. However, they are often so effective that their concentrations in fermentation broth might actually be quite low. Therefore, optimizing the fermentation conditions to obtain high yield of algicidal compound seems to be beneficial for both theoretical and applied researches. But incomplete information about the target chemical becomes the biggest obstacle to a successful optimization process, which requires reliable material quantification. This seems to be a paradox, and it might be partially responsible for the slow development of microbial algicides. Nevertheless, researchers have made some efforts to optimize the yield of algicidal microorganisms. The medium composition for the marine algicidal bacterium *Alteromonas sp.* DH46 were optimized using uniform design and the bacterial dry weight successfully increased by 107% and algicidal efficiency by nearly 10% [19]. Response surface methodology was used to obtain the best fermentation conditions for the algicidal bacterium R2, and the final cell density successfully increased without scarifying the algicidal rate [20]. However, these studies initially focused on the increase of biomass, which, theoretically speaking, has no absolute correlation with the yield of algicidal metabolites. The direct optimization for algicidal compound could be achieved when the chemical was well studied [21], but only few successful studies were reported when the chemical was unknown [22].

In recent years, more and more newly developed optimization strategies have been used in the fermentation industry. And the problems in optimizing the yield of algicidal compound seems solvable though the advanced artificial intelligence techniques with high efficiency and extensive application scope. One of these promising methods combines the use of artificial neural networks (ANNs) with genetic algorithms (GAs). An ANN is a computational model inspired by nervous systems and is capable of machine learning and output value prediction [23]. Its high accuracy in multi-factorial and nonlinear analysis makes it a good tool to simulate fermentation results. The GA is an optimization algorithm based on Darwinian evolution and Mendelism in order to carry out random, adaptive and parallel global searches [24]. Fully understanding the advantages of these two computational methods, many researchers couple GA with ANN to optimize fermentation conditions and obtain significant results [25]. Considering algicidal ratio shows positive but nonlinear correlation with the content of algicidal compound, ANN and GA seems to be the excellent tools to analyze and fit the data. However, there are still no reports concerning applying this method in the fermentation optimization of algicidal microorganisms.

An actinomycete strain *Streptomyces alboflavus* RPS [15], which was isolated from the sediment sample of Fujian Zhangjiangkou Mangrove National Nature Reserve, China, showed high algicidal activity against a typical harmful alga, *Phaeocystis globosa*. RPS lysed the algal cell by releasing an extracellular compound and the mycelial pellets were also capable of inhibiting algal growth in a seawater environment. To better understand its algicidal properties and prepare for the possible field test in future, we firstly tried to increase the production of mycelia and concentration of algicidal compound. In this new developed optimization protocol, we preferred to simplify the measurement of indexes, which took the dry mycelial weight as the biomass and algicidal ratio as the concentration of algicidal compound, to avoid unnecessary experimental errors. With the data obtained from single factor experiments and uniform design, we fully took the advantages of ANN and GA to fit the data and obtain the optimal medium compositions and cultivation conditions. And we finally verified the optimal fermentation conditions and compared the GA-ANN method with the traditional regression model.

## Results and discussion

### The effects of different nutrients and cultivation conditions on the growth of RPS

In order to optimize the fermentation conditions to increase the production of RPS, we should first understand which were the major influencing factors. More practically speaking, we should found out changing which nutrients or cultivation conditions would lead to an increased yield of biomass and algicidal compounds compared to the original fermentation conditions. Therefore, we set the control group as the baseline in order to make the changes caused by different nutrients and cultivation conditions more clearly comparable.

**Figure 1:**
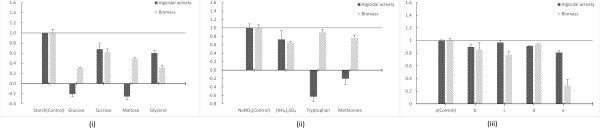
**The effects of different nutrients on the growth of RPS. (i)** Carbon sources. **(ii)** Nitrogen sources. **(iii)** Inorganic nutrients content. **a)** 0.5 g/L K_2_HPO_4_, 0.5 g/L MgSO_4_•7H_2_O, **b)** 0.75 g/L K_2_HPO_4_, 0.5 g/L MgSO_4_•7H_2_O, **c)** 0.25 g/L K_2_HPO_4_, 0.5 g/L MgSO_4_•7H_2_O, **d)** 0.5 g/L K_2_HPO_4_, 0.75 g/L MgSO_4_•7H_2_O, **e)** 0.5 g/L K_2_HPO_4_, 0.25 g/L MgSO_4_•7H_2_O.

Carbon and nitrogen sources are essential for the growth of microorganisms. Many microbes can utilize various carbon or nitrogen sources, but the morphologies and metabolites might be expressed in all sorts of ways. Based on the biomass results in Figures 1 (i) and (ii), we can see that even though all the carbon and nitrogen sources could support the growth of RPS, preferences for starch and NaNO_3_ showed clearly. The differences in the production of algicidal compounds were even more dramatic. The fermentation broth made up with glucose, maltose, tryptophan and methionine showed no algicidal activity, but on the contrary promoted the growth of algae. In summary, starch and NaNO_3_ were the most fit carbon and nitrogen sources for RPS fermentation, either in terms of biomass or algicidal activity. However, the most appropriate concentrations of these two nutrients require further studies.

Inorganic minerals also play a critical role in the lifecycle of microorganisms, although the requirement is much lower than that for a carbon and nitrogen source. In this study, we briefly tested the influence of different inorganic nutrient content on RPS. Judging by the biomass in Figure 1 (iii), the concentration of K_2_HPO_4_ and MgSO_4_ did not affect the growth of RPS very much, except for a 71.2% decrease caused by low MgSO_4_ content. Considering the algicidal activity, the distinctions were more minor, even the low biomass in the low MgSO_4_ situation only reduced the algicidal ratio by 18.9%. Interestingly, the middle inorganic concentration (0.5 g/L K_2_HPO_4_, 0.5 g/LMgSO_4_•7H_2_O), which acted as the control group, showed the highest biomass and algicidal activity, suggesting the importance of correct content of inorganics. Since the changes of these two inorganic minerals did not bring about higher yield of neither biomass nor algicidal compound, we would not take more effort to optimizing the inorganic mineral content for the moment.

**Figure 2:**
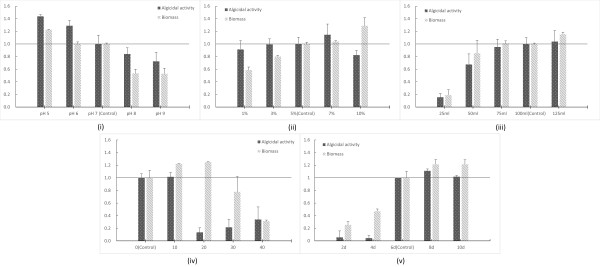
**The effects of different cultivation conditions inoculum size on the growth of RPS. (i)** Initial pH. **(ii)** Inoculum size. **(iii)** Loaded volume. **(iv)** Salinity values. **(v)** Fermentation time.

Every microbe has an optimum pH range. Most microorganisms are suited by a neutral environment while some are acidophilic or basophilic. The effect of initial pH on the fungus *Ganoderma lucidum*, which can produce simultaneously ganoderic acid and a polysaccharide, has been studied [26]. And the authors find that the maximum biomass and production of ganoderic acid is obtained at an initial pH of 6.5. However, the production of extracellular and intracellular polysaccharides becomes higher when the initial pH drops to 3.5. RPS lived better under a meta-acid environment (Figure 2 (i)). A low initial pH of 5 significantly increased the biomass and algicidal activity by 22.5% and 43.8%, respectively. This large improvement with low initial pH suggested that more thorough studies should be conducted.Inoculum size strongly affected the growth rate of the strain. High inoculum size could bring forward the stationary phase and the synthesis of metabolites, therefore also decrease the possibility of contamination. However, too high an inoculum size might also reduce the yield of products owing to the high consumption of oxygen. In Figure 2 (ii), the biomass had a positive correlation with inoculum size while the algicidal activity stayed at a high level even with the lowest inoculum size of 1%. Interestingly, the 10% inoculum size raised the biomass by 28.7%, but the algicidal activity decreased by 26.6% under the same inoculum size. This could be explained by the early coming of the late stationary phase blocking the synthesis of algicidal compounds. Thus, further optimization seemed to be necessary.In most cases, the loaded volume affected the fermentation process owing to its association with dissolved oxygen. Lower loaded volume led to a higher oxygen transfer coefficient under the same shaking speed. In Figure 2 (iii), the biomass and algicidal activity is raised along with the volume up to 75 mL, and no huge gap is seen between 75, 100 and 125 mL. This result indicated that the high oxygen level might be a restricting factor to the growth of RPS.RPS was isolated from the sediment sample of an estuarine area, which explained why its biomass could reach a peak value at a salinity of 20 (124.8% compared to the control group in Figure 2 (iv)). However, the algicidal activity showed a different pattern. Only salinity levels of 0 and 10 induced the production of algicidal compounds, compared to the most fit salinity of 20 for mycelia growth. A good fermentation result under 0 salinity was beneficial for future large-scale production since high salinity has a strong corrosion effect on steel fermentation tanks.Fermentation time can characterize the growth rate of a strain, and RPS was a relatively slow-growing microbe (Figure 2 (v)). The biomass continued to increase even after 8d, but the algicidal compounds were secreted only after 6d, which might mark the beginning of the stationary phase. The slight decrease of the algicidal ratio at 10d also confirmed the situation in the case of high inoculum size, suggesting the importance of harvesting the fermentation broth at an appropriate growth phase in order to maximize the yield of algicidal compounds.

In summary, there were five factors that increased the production of RPS. Two of them (salinity and loaded volume) were not suitable for future large-scale fermentation conditions. Considering the importance of carbon and nitrogen content, five factors were used for the more detailed multi-factorial optimization: starch content, sodium nitrate content, inoculum size, initial pH and fermentation time.

### Uniform design and regression model

A uniform design seeks design points that are representative and uniformly scattered on the domain [27]. Therefore, we could achieve the same goal as other statistical design methods, such as orthogonal design, with fewer experimental groups [19,24]. Here we used the Data Processing System (Version 7.05) for the experimental design and subsequent data analysis along with the generation of regression models. The results from the different experimental groups are presented in Table 1.

**Table 1:** Uniform-design-table and the results

**Exp. no.**	**Factors**					**Indexes**	
	**X1**	***X*** **2**	**X3**	**X4**	**X5**	**Y1**	**Y2**
	**Starch (g/L)**	**NaNO**_**3 **_**(g/L)**	**Inoculum size (%)**	**Initial pH**	**Time (h)**	**Algicidal ratio (%)**	**Dry mycelial weight (g/100mL broth)**
N1	16	0.3	4.5	5	108	89.5686%	0.05715
N2	17	1.4	10.5	9	168	76.9796%	0.1079
N3	11	0.4	7.5	10	204	81.6116%	0.08085
N4	19	1.5	8	4	96	81.0957%	0.05875
N5	12	1.7	7	5.5	192	79.7349%	0.12175
N6	15	1	9.5	4.5	228	84.5514%	0.2283
N7	20	0.9	6.5	9.5	60	8.3172%	0.03545
N8	9	0.8	6	3.5	156	12.5545%	0.0001
N9	18	0.7	3.5	8	180	86.3218%	0.11275
N10	8	1.1	8.5	8.5	120	84.6537%	0.0412
N11	14	1.6	5	10.5	132	76.7769%	0.04345
N12	21	1.2	5.5	7	216	74.7522%	0.1718
N13	10	1.3	4	6.5	72	67.7670%	0.02315
N14	13	0.6	10	7.5	84	77.7472%	0.04345
N15	22	0.5	9	6	144	80.3603%	0.12165

Based on the results of dry mycelial weight, a multiple regression equation was generated: Y2 = -0.543313758 + 0.022952473674X1 + 0.05158971648X3 + 0.08619420430X4-0.0013331394535X5-0.0004410784967X1*X1-0.0013657027343X3*X3-0.004819912252X4*X4-0.0016301914750X1*X3 + 0.00030259683893X1*X4 + 0.000030318409839X1*X5-0.004189833135X3*X4 + 0.00020227979933X3*X5 + 0.000021989005098X4*X5. The correlation indexes were as follows: *R* = 1.0000, *F* value =38461.4808, *p* = 0.0040, Durbin-Watson value =2.17768003. The optimal fermentation conditions for a maximum biomass of 0.2598 g/100 mL were: 16.31 g/L starch, 0.52 g/L NaNO_3_, inoculum size 10.5%, initial pH 5.41, and fermentation time 228 h.

Also, based on the results of the algicidal ratio, another multiple regression equation was generated: Y1 = -3.97848306 + 0.31068222407X1 + 0.6327387394X4 + 0.006103641490X5-0.005977938516X1*X1 + 0.0021886562363X2*X2-0.022509013327X4*X4-0.000031536339359X5*X5-0.0019135332295X1*X3-0.020236820212X1*X4 + 0.00015411255817X1*X5-0.0027135967924X3*X4 + 0.00030307344603X3*X5-0.00006432940808X4*X5. The correlation indexes were as follows: R = 1.0000, F value = 38461.4808, p =0.0040, and Durbin-Watson value = 1.87922745. The optimal fermentation conditions for highest algicidal activity of 103% were: 18.07 g/L starch, 1.7 g/L NaNO_3_, inoculum size 3.5%, initial pH 5.50, and fermentation time 152 h.

### Determination of the structure of artificial neural networks

The first step to build a neural network is to determine its structure, including the input neurons, the output neurons, the hidden neurons, and the training algorithm. The input and output neurons were consistent with the original experimental data. We also chose a back-propagation algorithm, which is commonly used in the fermentation industry, to train the network. However, the number of neurons in the hidden layer requires more calculation to minimize the error. Too few hidden neurons would lower the precision of the neural network, but too many might deviate the model from the real circumstance so wing to counting in some data undulation caused by experimental error. Therefore, we determined the appropriate number of hidden neurons firstly (Table 2). In the case of dry mycelial weight, the training error dropped to a relatively low level when the number of hidden neurons reached nine. Even though the prediction error did not show a similar pattern, we could easily see that nine hidden neurons had the highest prediction accuracy. Thus, we determined the structure of the neural network for dry mycelial weight as 5-9-1. In the case of the algicidal ratio, a number of hidden neurons above nine also decreased the training error to a low level. However, the minimum prediction error appeared only after the number of hidden neurons was 12, and so the structure of the neural network for algicidal ratio was determined as 5-12-1.

**Table 2:** Error of the artificial neural network with different numbers of hidden neurons

**Number of hidden neurons**	**Dry mycelial weight**		**Algicidal ratio**	
	**Training error**	**Prediction error**	**Training error**	**Prediction error**
3	0.011	0.05911	0.08859	0.53504
4	0.0024	0.08869	0.02129	0.66232
5	0.00212	0.05847	0.00561	0.6092
6	0.00414	0.06218	0.00556	0.50672
7	0.00438	0.06918	0.00523	0.40763
8	0.00377	0.06369	0.01004	0.54822
9	0.0018	0.05719	0.00412	0.40388
10	0.00186	0.06579	0.0047	0.44591
11	0.00216	0.07613	0.00467	0.46936
12	0.002	0.07142	0.00448	0.31169

### Optimization of artificial neural networks using the genetic algorithm

The precision of an ANN is greatly affected by the initial weights and thresholds of the network, and so we applied the GA, which used the sum of training error as the fitness, to seek the best weights and thresholds. The processes of optimization and the precision of the optimized neural networks are shown in Additional file 1: Figures S1 and S2. There is no doubt that the high accuracy of these neural networks promised good simulation of fermentation and therefore increased the possibility of gathering convincing results by further optimization.

### Genetic algorithm for best fermentation conditions

**Figure 3:**
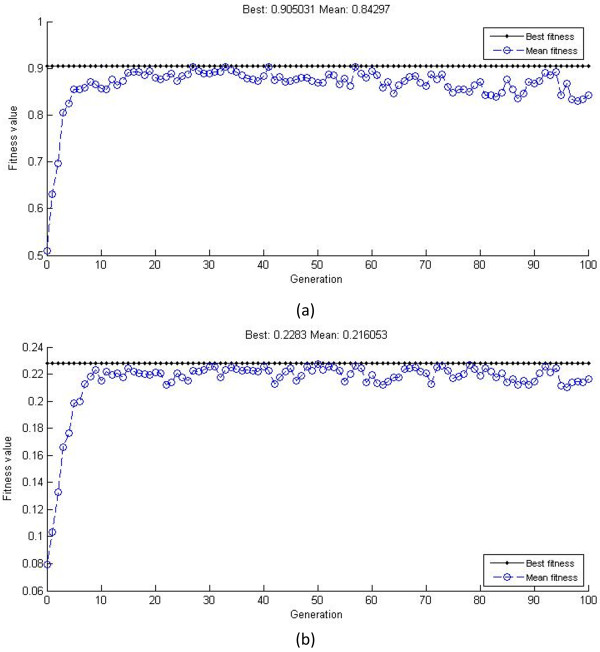
**Fitness curves for the optimization of neural networks. (a)** Optimization for algicidal ratio. **(b)** Optimization for dry mycelial weight.

One of the advantages of GA is that it does not require a specific objective function, which expands its applications largely, and so we used it again to obtain the best medium composition and cultivation conditions based on the neural networks. Figure 3 shows the optimization processes for each neural network. The optimal fermentation conditions were as follows: 19.93 g/L starch, 0.66 g/L NaNO_3_, inoculum size 9.2%, initial pH 5.20, and fermentation time 216 h for a maximum dry mycelial weight of 0.2283 g/100 mL; 17.76 g/L starch, 1.59 g/L NaNO_3_, inoculum size 8.1%, initial pH 5.23, and fermentation time 185 h for the highest algicidal ratio of 90.5%.

### Verification experiments

No matter how wonderful the results for the mathematic models are, experimental results are the final judges. As shown in Table 3, the optimization effects of both models were quite similar, which was not a big surprise because of the similar biases of nitrogen source, initial pH and fermentation time. The optimal fermentation conditions greatly increased the RPS biomass by 66.30% for uniform design and 69.27% for the GA-ANN method. The algicidal activity was also enhanced, although the degrees of growth were much smaller because of the high algicidal ratio of the original. However, the neural networks showed their improvement for a much higher prediction accuracy than the regression models (1.79 to 16.27%, and 5.54 to 22.14%). Moreover, the extremes that came up with the regression model (inoculum size and fermentation time) implied its limited ability of optimization under complicated circumstances.

**Table 3:** Verification of optimal fermentation conditions

**Indexes**		**Regression model**	**GA-ANN method**	**Control**
Algicidal ratio	Experimental results	88.59 ± 3.46%	92.15 ± 1.12%	78.83 ± 2.76%
	Improvement compared to control	12.38%	16.90%	/
	Prediction	103.00%	90.50%	/
	Prediction error	16.27%	1.79%	/
Dry mycelial weight	Experimental results	0.2127 ± 0.0191 g	0.2165 ± 0.072 g	0.1279 ± 0.0116 g
	Improvement compared to control	66.30%	69.27%	/
	Prediction	0.2598 g	0.2283 g	/
	Prediction error	22.14%	5.45%	/

## Conclusions

In this study, we innovatively combined the use of uniform design with ANN coupling GA in the optimization of the fermentation conditions of an algicidal actinomycete, and reflected the efficiency of uniform design, the ‘eurytopicity’ and accuracy of GA and BP-neural network, which overcame the quantitative problem of algicidal compound. The further application of algicidal microorganisms also became more plausible. Despite the fact that more and more researchers focus on various genetic modification methods to boost the productivity of microorganisms, fully developing the potential of the original strain by optimizing the fermentation conditions is still a more economic, fast and environmentally safe way especially in the field of algicidal preparations that require more thorough theoretical studies.

In many studies, multi-factorial analysis was used in the optimization of medium composition. However, the importance of some nutrients might not be well quantified because of their concentrations (such as K_2_HPO_4_ in this study), while cultivation conditions (such as inoculum size, initial pH and fermentation time in this study) can play more critical roles and also interact with the medium composition. For example, inoculum size affects the growth rate of the strain, which is important to some slow-growing microorganisms and also directly connected to the consumption rate of the nutrients. Thus, in our opinion, applying single factor experiments to decide the important factors was necessary before proceeding to multifactorial optimization.

Wisely applying these multi-factorial analytical methods was even more important. In this study, we successfully saved the use of many experimental groups, thanks to the advantages of uniform design. Nevertheless, the traditional quadratic polynomial stepwise regression method showed its limitations in simulating the fermentation, the result of which were even absurd in the case of the algicidal ratio with nonlinear variation(>100%). The ANN and GA seemed to be much more convincing based on our final verification experiments. However, it is undeniable that such great outcomes were based on the brilliant experimental sets coming from uniform design, and we should rationally choose and combine the algorithms, and then use their advantages to achieve our goals.

Beside the challenges during the development of microbial algicides, establishing a comprehensive theoretical system to guide the application of algicidal microorganisms is another difficulty that we have to face. In recent years, more and more researchers focused on the interaction mechanism between algae and microorganisms. Just like RPS, many algicidal microbes inhibit the growth of harmful algae or cause the lysis of algal cells by secreting some biological active compounds, which shares quite a lot of similarities with alleopathy. Many studies revealed the fact that these compounds would lyse the cells by inducing oxidative stress and destroying the photosynthetic system [2,28]. Another important red tide control microbial factor is virus. Early in 1963, algal viruses had been isolated and identified [29]. With the gradual understanding of the crucial roles that algal virus plays in marine environment [30], virus also becomes a potential candidate for algal bloom control owing to their high efficiency and species-specificity. Except for the viruses, a newly found pathogen, which was identified as the protist *Pseudobodo* sp. and could directly attack the algal cells, largely expanded our research and application prospect for algicidal microorganisms [31]. There is no doubt that microorganisms will be the key players in future red tide control [32], and the ongoing theoretical researches will serve the mature of field applications.

In a word, this study provided a clear way to optimize the fermentation conditions of a novel algicidal actinomycete, and also laid the foundation for the development of algicidal preparations in the future.

## Methods

### Algal culture and evaluation of algal biomass

The *Phaeocystis globosa* culture was obtained from the State Key Laboratory of Marine Environmental Science (Xiamen University). The culture was maintained in sterilized f/2 medium under a 12 h: 12 h light-dark cycle with a light intensity of 4000 lx at 20 ± 1°C. When evaluating algal biomass, the *P. globosa* culture was transferred to a 24-well cell plate and the fluorescent intensity (RFU) measured under an excitation wavelength of 440 nm and emission wavelength of 680 nm (Spectra max M2, Molecular Devices Corporation). Earlier study has confirmed that this is a convenient and accurate method to evaluate biomass [33].

### Isolation and cultivation of *Streptomyces alboflavus* RPS

The strain was isolated from a sediment sample in the Fujian Zhangjiangkou Mangrove National Nature Reserve, China, through the dilution plating procedure with modified Gause medium (soluble starch 15 g/L, NaNO_3_ 1 g/L, K_2_HPO_4_ 0.5 g/L, MgSO_4_•7H_2_O 0.5 g/L, FeSO_4_•7H_2_O 0.01 g/L, dissolved in natural seawater for agar plates, but dissolved in deionized water for liquid fermentation). The colony was then purified several times and identified as *Streptomyces alboflavus* based on its physiological and biochemical characteristics and 16S rDNA sequencing. The strain was stored at -80°C in 10% (v/v) glycerol and inoculated into liquid medium (5%, v/v) for further study in a rotary shaker (28°C, 200 rpm) for 6d until the mycelia turned yellow-red. The growth of RPS was estimated using biomass and algicidal activity. The mycelia were collected by filtered and dried for 3 d to a constant weight as the biomass, and algicidal activity was evaluated by inoculating 200 μL of RPS culture into 1.8 mL of logarithmic phase algal culture (RFU ≈ 300, 2.42 × 10^6^ cells/mL), while 200 μL of fresh medium was added to the algal culture as a control. The algicidal ratio was then calculated using the following formula:

$$\text{Algicidal}\;\text{ratio}\;\left(\%\right)=\frac{F_{0}‒F_{t}}{F_{0}}\times 100,$$

where F_t_ is the fluorescent intensity of the treated algal culture, and F_0_ the fluorescent intensity of the control group.

All shaken flask experiments included at least two parallel samples.

### The effects of different nutrients and cultivation conditions on the growth of RPS

We used single factor experiments, meaning only one of the nutrients or cultivation conditions was changed in each experimental group and the other influencing factors remained the same as the original. The setups of each experimental group were shown below.

Carbon source: starch, glucose, sucrose, maltose and glycerol.

Nitrogen source: tryptophan, methionine, sodium nitrate, and ammonium sulfate.

Inorganic content: a) 0.5 g/L K_2_HPO_4_, 0.5 g/L MgSO_4_•7H_2_O, b) 0.75 g/L K_2_HPO_4_, 0.5 g/L MgSO_4_•7H_2_O, c) 0.25 g/L K_2_HPO_4_, 0.5 g/L MgSO_4_•7H_2_O, d) 0.5 g/L K_2_HPO_4_, 0.75 g/L MgSO_4_•7H_2_O, e) 0.5 g/L K_2_HPO_4_, 0.25 g/L MgSO_4_•7H_2_O.

Initial pH: 5, 6, 7, 8 and 9.

Inoculum size: 1, 3, 5, 7 and 10%, v/v.

Loaded volume: 25, 50, 75, 100 and 125 mL in 250 mL flasks. The biomasses were converted into 100 mL for comparison.

Salinity: 0, 10, 20, 30 and 40‰.

Fermentation time: 2, 4, 6, 8 and 10 d.

To better quantify the influence of each factor and eliminate the minor errors caused by different experiment batches, we normalized the experimental results of the control group (which used the same cultivation conditions as in the origin experiment) to 1 and the experimental results of the other groups were compared to that of the control group to show the differences.

### Uniform design for multifactor optimization

Based on the single factor experiments above, we brought the five main influencing factors, which were starch content, sodium nitrate content, inoculum size, initial pH and fermentation time, to the next step - multifactor optimization. The Data Processing System (DPS Version 7.05) was used to generate the experimental design, statistical analysis and regression model using the quadratic polynomial stepwise regression method. Based on the uniform design table U_15_(15^5^), 15 experimental groups with the five independent variables (X1, *X*2, X3, X4 and X5) were set for testing the two dependent variables, Y1 (algicidal ratio) and Y2 (dry mycelial weight). Details concerning the experimental design and results are shown in Table 1. Two regression models were obtained for Y1 and Y2, followed by the acquisition of the optimal combination of cultivation conditions for the growth of RPS.

### Combination of the artificial neural network and the genetic algorithm

The Matlab R2013a software was used for ANN modeling and GA optimization. In this study, two separate neural network models were constructed to model the fermentation process and predict the biomass and algicidal activity. We used the data from uniform design as the training samples. Thus, each neural network consisted of five input neurons (starch content, sodium nitrate content, inoculum size, initial pH and fermentation time) and a single output neuron (dry mycelial weight or algicidal ratio). The optimization process was made up of three steps.

1) We tested the error with different neurons in a hidden layer to determine the best structure of the neural network. Based on experience and literature [34,35], we primarily chose three to 12 hidden neurons to conduct the error calculation. The number of neurons in the hidden layer was determined taking into account of two types of error, training error and prediction error. The program randomly picked up 13 experimental groups as the training samples, and the other two groups as the test samples. The neural networks were trained with different numbers of hidden neurons and the simulated results were compared to training and test samples and the 2-norm of training and prediction errors further worked out. Considering the influence of initial weights and thresholds in the neural network and the experimental error of the samples, we replicated the calculation 10 times.

2) We applied the GA to optimize the weights and thresholds of back-propagation (BP)-neural networks to increase their accuracy. To fully simulate the fermentation process, we used all 15 experimental samples to train the ANN. The max epoch was set as 2000 and the training goal was 1 × 10^-6^. In the GA, the sum of training errors of each sample (absolute value) was defined as the fitness. The program sought the minimum fitness using the procedure of selection, crossover and mutation. The other parameter settings were as follows: maxgen = 50, sizepop = 20, Pcross = 0.8, Pmutation = 0.05.

3) With the optimal neural networks, we applied the GA again to seek the best fermentation conditions for RPS. In this step, the simulation result of the neural network was defined as the fitness. The algorithm sought the maximum fitness with the following parameter settings: maxgen = 100, sizepop = 80, Pcross = 0.4, Pmutation = 0.05.

### Verification experiments

Finally, we verified the four groups of optimal fermentation conditions through experiments and compared the final results and accuracy of the two prediction models (regression model and neural network model).

## Competing interests

The authors declare that they have no competing interests.

## Authors’ contributions

GC designed the study, carried out most of the experiments, established the artificial neural network and drafted the manuscript. WZ helped to design the study and participated in the statistical analysis. XY designed the uniform design experiments, participated in the measurements of biomass and algicidal ratio and helped the statistical analysis. BZ primarily studied the strain and developed the method of determining the algicidal ratio. TZ gave the scientific ideas and guidance, directed and conceived of the whole study, and provided the experimental platform and financial support. All authors read and approved the final manuscript.

## Supplementary Material

Additional file 1: Figure S1 Optimization of neural network for dry mycelial weight using a genetic algorithm. (a) Fitness curve of the genetic algorithm. (b) Prediction error of the network for each training sample. **Figure S2.** Optimization of the neural network for the algicidal ratio using a genetic algorithm. (a) Fitness curve of the genetic algorithm. (b) Prediction error of the network for each training sample. Click here for file
