# Supplementary material for: Combination of uniform design with artificial neural network coupling genetic algorithm: an effective way to obtain high yield of biomass and algicidal compound of a novel HABs control actinomycete
Source: Microb Cell Fact. 2014 May 24;13:75. doi: 10.1186/1475-2859-13-75 (PMC4051378; doi:10.1186/1475-2859-13-75)
Supplement: Additional file 1: Figure S1 — Optimization of neural network for dry mycelial weight using a genetic algorithm. (a) Fitness curve of the genetic algorithm. (b) Prediction error of the network for each training sample. Figure S2. Optimization of the neural network for the algicidal ratio using a genetic algorithm. (a) Fitness curve of the genetic algorithm. (b) Prediction error of the network for each training sample. [file 1475-2859-13-75-S1.docx]

**Supplementary data**

Manuscript title: Combination of Uniform Design with Artificial Neural Network Coupling Genetic Algorithm: An Effective Way to Obtain High Yield of Biomass and Algicidal Compound of a Novel HABs Control Actinomycete

Authors: Guanjing Cai^1, 2‡^, Wei Zheng^1‡^, Xujun Yang^1^, Bangzhou Zhang^1^, Tianling Zheng^1, 2^*

Number of figures: 2

Number of tables: 0

Number of pages: 3


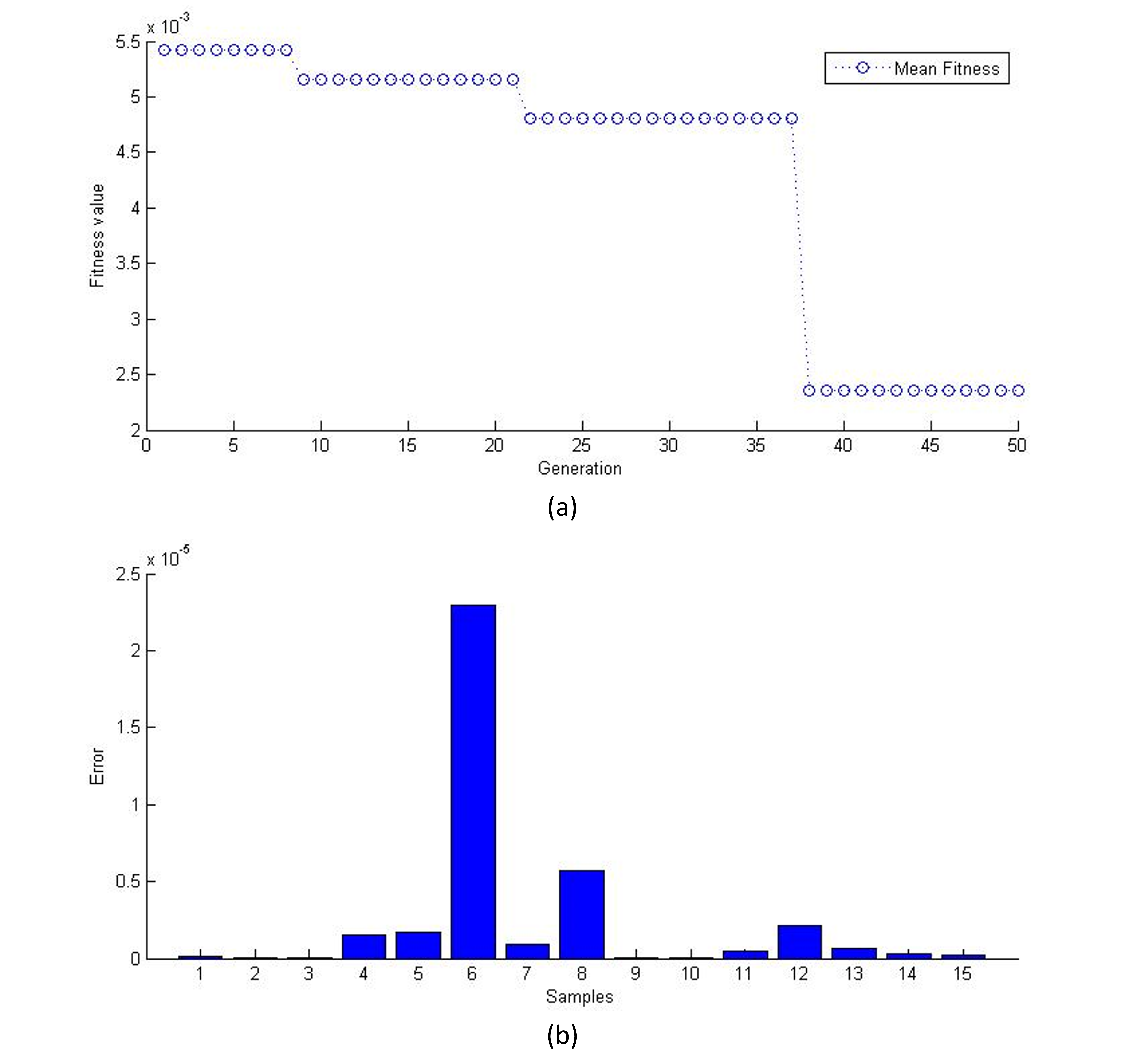


**Figure S1.** Optimization of neural network for dry mycelial weight using a genetic algorithm. (a) Fitness curve of the genetic algorithm. (b) Prediction error of the network for each training sample.


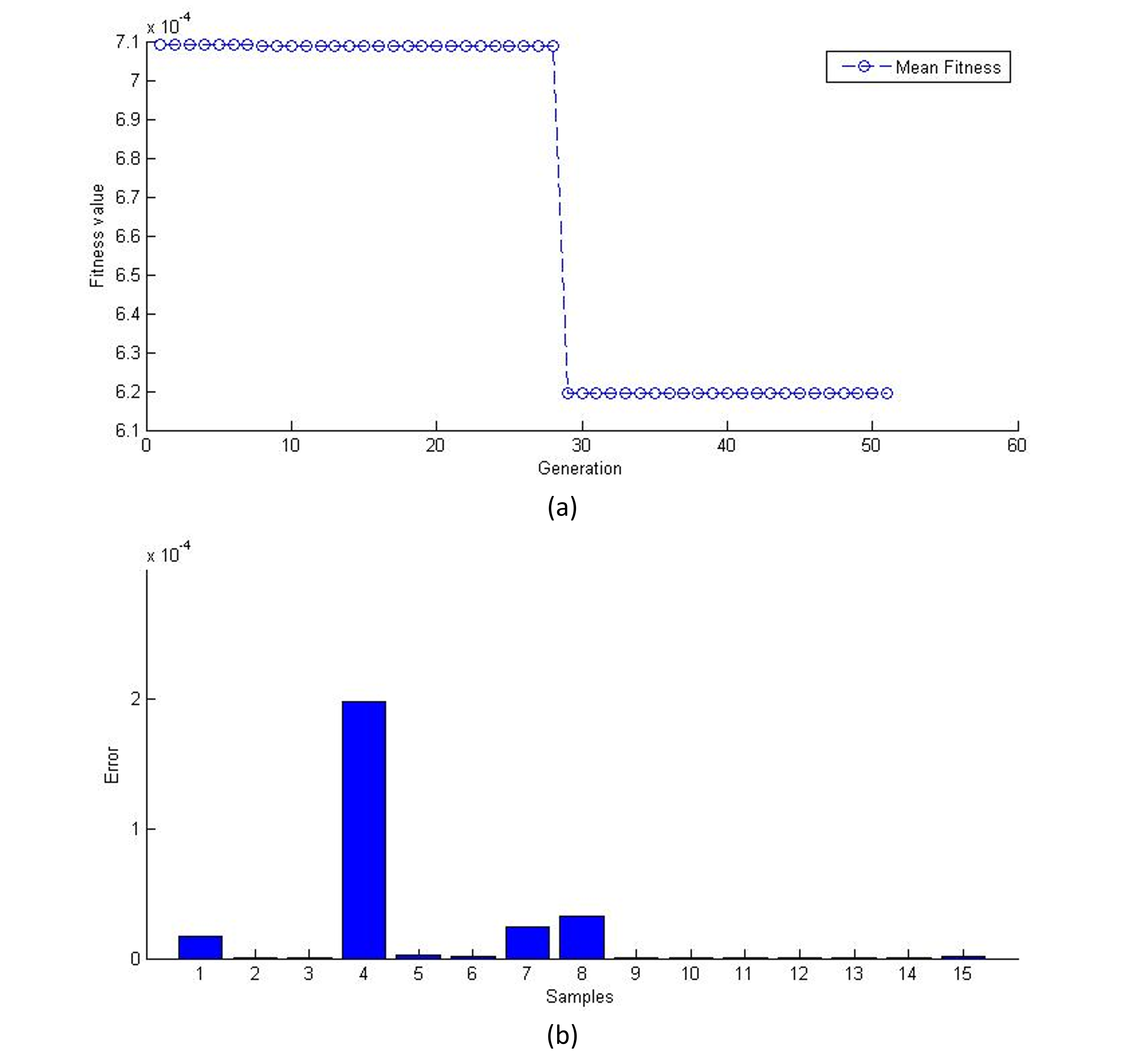


**Figure S2.** Optimization of the neural network for the algicidal ratio using a genetic algorithm. (a) Fitness curve of the genetic algorithm. (b) Prediction error of the network for each training sample.
